# Avascular femoral necrosis as part of Cushing syndrome presentation: a case report 

**DOI:** 10.1186/s13256-021-02882-7

**Published:** 2021-05-26

**Authors:** Daniela Salazar, César Esteves, Maria João Ferreira, Jorge Pedro, Tiago Pimenta, Raquel Portugal, David Carvalho

**Affiliations:** 1 Department of Endocrinology, Diabetes and Metabolism, Centro Hospitalar Universitário de São João, 4200-319 Porto, Portugal; 2 Faculty of Medicine of University of Porto, Porto, Portugal; 3 Institute for Research and Innovation in Health, University of Porto, Porto, Portugal; 4 Department of Surgery of Centro Hospitalar, Universitário de São João, Porto, Portugal; 5 Department of Pathology of Centro Hospitalar, Universitário de São João, Porto, Portugal

**Keywords:** Avascular bone necrosis, Cushing syndrome, Adrenal adenoma, Case report

## Abstract

**Background:**

The clinical characteristics and complications of Cushing syndrome (CS) are well known and described in the literature. Nevertheless, rare, atypical presentations may go unnoticed. Osteonecrosis is a well-documented complication of glucocorticoid therapy. However, endogenous hypercortisolism is a rare, but relevant, cause of bone avascular necrosis. We describe the case of a woman with CS undiagnosed for 2 years after presenting with femoral avascular necrosis.

**Case presentation:**

A 38-year-old Caucasian woman was referred for evaluation of secondary amenorrhea, associated with oral contraception withdrawal in the context of deep venous thrombosis (DVT). She had a previous right hip arthroplasty for treatment of avascular necrosis of the femoral head, diagnosed after 3 years of progressive right hip pain and limited mobility. She also had high blood pressure (HBP) of 5 years’ duration, and reported weight gain (4 kg in 2 years). There was no history of infertility (gravida 2, para 2). Physical examination revealed buffalo hump, truncal obesity, facial plethora, muscular atrophy and proximal myopathy, and easy bruising (under anticoagulant treatment for DVT). Workup showed abnormal overnight dexamethasone suppression test (DST) (serum cortisol 21.5 µg/dL; normal < 1.8 µg/dL), elevated 24-hour urinary free cortisol (UFC) (728.9 µg/day; reference range 36.0–137.0 µg/day), and suppressed plasma adrenocorticotropic hormone (ACTH) (< 1.0 pg/mL), findings consistent with ACTH-independent CS. Urinary metanephrines and catecholamines were normal, and the remaining analytical study showed no major changes, apart from glycated hemoglobin (HbA1c) of 6.8%. Adrenal computed tomography (CT) scan showed a 25 mm lesion in the left adrenal gland, with density non-suggestive of adenoma. The patient underwent unilateral adrenalectomy and started steroid replacement. Histology revealed an adrenal cortex adenoma. Three months after surgery the patient presented with resolution of HBP and hypercortisolism (UFC 37.4 µg/day; reference range 36.0–137.0 µg/day).

**Conclusion:**

In some cases, CS signs may go unnoticed and the diagnosis postponed. Avascular necrosis is a rare presenting feature of endogenous hypercortisolism, and, if left untreated, complete collapse of the femoral head may ensue, rendering the need for hip replacement in up to 70% of patients. Suspicion and recognition of atypical features is therefore important in avoiding complications and delay in treatment of CS.

## Background

Osteonecrosis is a pathological process characterized by cellular death of bone and joint components due to interruption of the blood supply, which results in structural collapse and painful loss of joint function, and has been associated with numerous conditions and therapeutic interventions [1]. Glucocorticoid use is reported to contribute to 10–30% of all cases of avascular necrosis of the femoral head; however, only a few cases are described as presentation of endogenous hypercortisolism, mostly in the context of Cushing syndrome (CS) [2–12].

We describe the case of a young woman with adrenocorticotropic hormone (ACTH)-independent CS that was undiagnosed for 2 years after presenting with avascular necrosis of the right hip without any apparent cause, which was ultimately treated with hip arthroplasty.

## Case presentation

**Fig. 1:**
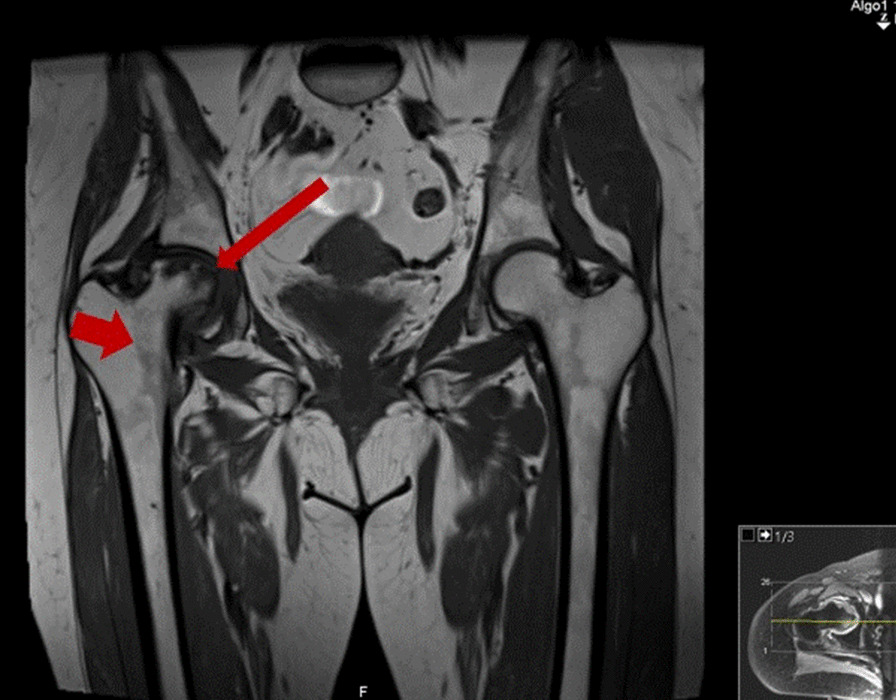
Magnetic resonance imaging (MRI) of the hip. Small to moderate articular effusion in the right hip joint, with synovitis and marked dysmorphia of the femoral head with flattening of its superior slope (*thin red arrow*), and peripheral, subcortical edema of the femoral head involving almost all of the articular surface, delimited by a well-defined serpiginous line (*thick red arrow*). In the context of the given patient, these findings should represent avascular necrosis of the femoral head, probably post-traumatic, in stage IV

A 38-year-old Caucasian woman was referred to our department in November 2017 for evaluation of secondary amenorrhea. Absence of menstrual cycle followed oral contraception withdrawal in the context of spontaneous deep venous thrombosis (DVT) in 2015. Menarche was at the age of 13, and menstrual cycles were regular before the beginning of oral contraceptives. The patient had no previous history of infertility (two pregnancies and two deliveries) and reported weight gain of about 4 kilograms in the previous 2 years. She denied any symptoms of hyperandrogenism or virilization such as acne, hirsutism, or voice deepening. She also had a history of high blood pressure (HBP) for the previous 5 years, but no history of diabetes. At the time of initial evaluation, she was medicated with nebivolol/hydrochlorothiazide 5/25 mg once a day, rivaroxaban 20 mg once a day, and aceclofenac 100 mg twice a day. The patient complained of progressive and persistent right hip pain that started around 4 months after her second pregnancy and continued for 3 years, and was associated with limited mobility. Hip magnetic resonance imaging (MRI) study revealed avascular necrosis of the right femoral head (Fig. 1), and led to right hip arthroplasty 1 year before endocrinology evaluation. Although mobility was recovered after surgery, the patient continued to complain of proximal muscle weakness. She denied relevant family medical history. Physical exam showed a body mass index (BMI) of 25.96 kg/m^2^, facial plethora, truncal obesity, and supraclavicular fat pads. Muscular atrophy and easy bruising were apparent. No skin striae, hirsutism, or acne were seen.

Our workup revealed serum cortisol of 21.5 µg/dL after overnight dexamethasone suppression test (DST) (normal < 1.8 µg/dL), elevated 24-hour urinary free cortisol (UFC) (728.9 µg/day; normal 36.0–137.0 µg/day), and suppressed morning plasma ACTH (< 1.0 pg/mL; normal < 63.3 pg/mL), findings consistent with ACTH-independent CS. She had normal blood count in liver and kidney function tests, and glycated hemoglobin (HbA1c) of 6.8%. Electrolytes were normal, with the exception of slightly decreased phosphorus (2.3 mg/dL; reference range 2.7–4.5 mg/dL). The remaining hormonal study showed mild changes compatible with pituitary function suppression from excess cortisol (Table 1).

**Table 1:** Endocrine assessment baseline

Test	Result	Reference range
Cortisol (after 1 mg overnight DST)	**21.5 µg/dL**	< 1.8 µg/dL
ACTH	**<1.0 p/mL**	< 63.3 pg/mL
24-hour UFC	**728.9 µg/day**	36.0–137.0 µg/day
TSH	0.86 µUI/mL	0.35–4.94 µUI/mL
FT4	0.76 ng/dL	0.70–1.48 ng/dL
FT3	**1.53 pg/mL**	1.71–3.71 pg/mL
IGF-1	119 ng/mL	57–241 ng/mL
PRL	11.9 ng/mL	4.8–23.3 ng/mL
FSH	4.44 mUI/mL	2.40–12.60 mUI/mL (follicular phase)
LH	0.53 mUI/mL	3.50–12.50 mUI/mL (follicular phase)
Estradiol	10.4 pg/mL	12.5–166 pg/mL (follicular phase)
SHBG	**12.7 nmol/L**	26.1–110.0 nmol/L
Total testosterone	**< 0.03 ng/mL**	0.06–0.82 ng/mL
DHEA-S	**11.3 µg/dL**	60.9–337.0 µg/dL
δ-4-androstenedione	< 0.3 ng/mL	0.30–3.30 ng/mL
17-OH-Progesterone	0.24 ng/mL	0.6–2.6 ng/mL (follicular phase)

*ACTH* adrenocorticotropic hormone, *DHEA-S* dehydroepiandrosterone sulfate, *FSH* follicle-stimulating hormone, *FT3* free triiodothyronine, *FT4* free thyroxine, *IGF-1* insulin-like growth factor 1, *LH* luteinizing hormone, *PRL* prolactin, *SHBG* sex hormone-binding globulin, *TSH* thyroid-stimulating hormone, *UFC* urinary free cortisol. Abnormal values are presented in bold

**Fig. 2:**
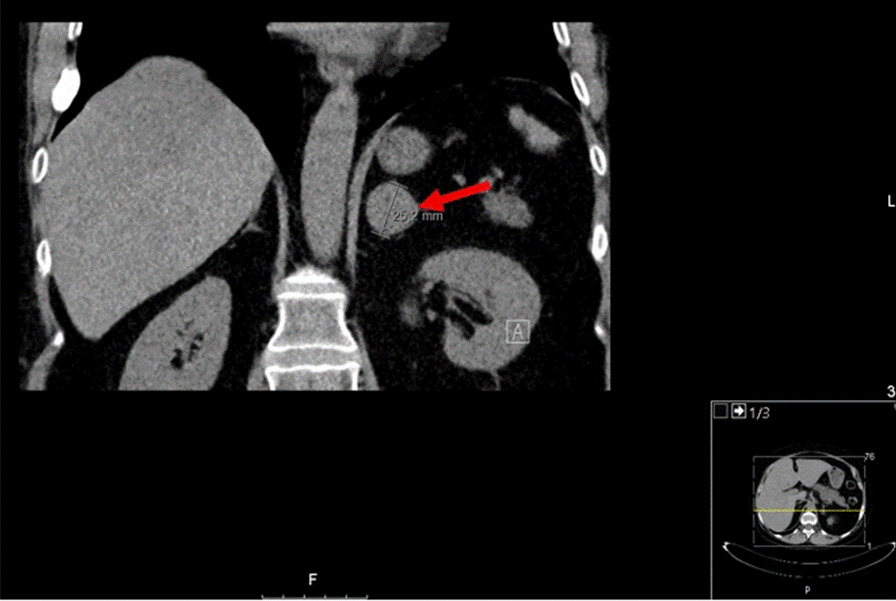
Computed tomography (CT) scan showing a 2.5 mm adenoma of the left adrenal gland (red arrow)

The patient underwent adrenal computed tomography (CT) scan, which showed a 25 mm lesion in the left adrenal gland, with density non-suggestive of adenoma (Fig. 2). She was promptly scheduled for unilateral adrenalectomy and was started on steroid replacement therapy after surgery.

Three months after surgery she presented with resolution of HBP and recovered regular menstrual cycle; thyroid function (thyroid-stimulating hormone 2.94 µUI/mL [reference range 0.35–4.94 µUI/mL], thyroxine 0.75 ng/dL [reference range 0.70–1.48 ng/dL], triiodothyronine 3.31 pg/mL [1.71–3.71 pg/mL]) and UFC (37.4 µg/day [reference range 36.0–137.0 µg/day]) were normal. Histological evaluation of the excised adrenal gland revealed an adrenal cortex adenoma. Bone densitometry was compatible with osteoporosis in the lumbar column (Z-score −2.2, T-score −2.3) and severe osteopenia in the proximal femur (Z-score −2.4, T-score −2.5). She was started on alendronate 70 mg/day and cholecalciferol 600 IU daily to reduce fracture risk.

**Fig. 3:**
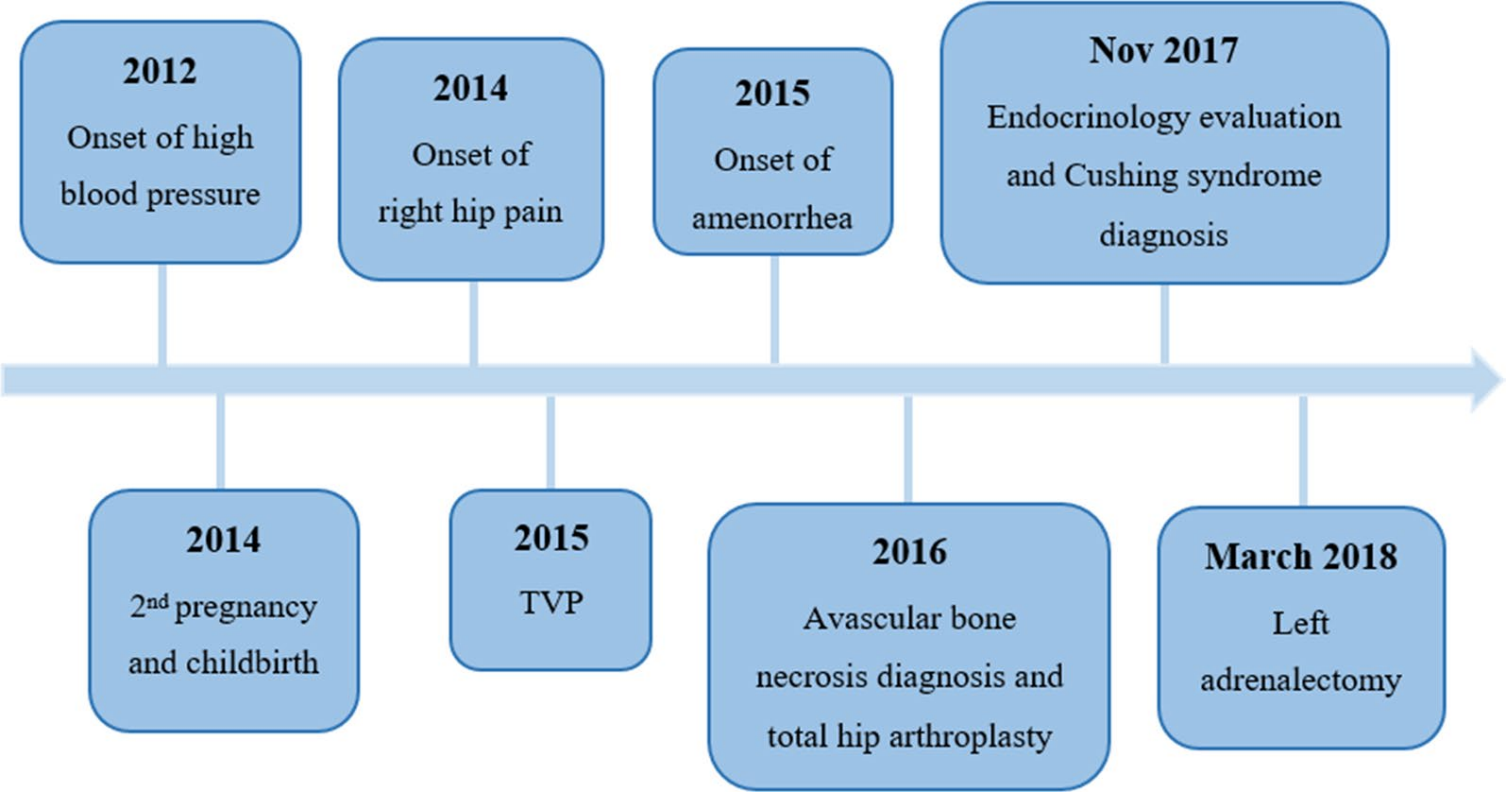
Case report organized timeline

Ten months after adrenalectomy, she underwent an ACTH stimulation test. Serum cortisol was 0.2 µg/dL at baseline and 1.5 µg/dL after 60 minutes, revealing adrenal insufficiency, requiring postponement of hydrocortisone withdrawal (she is currently treated with hydrocortisone 7.5 mg in the morning + 5 mg at lunch + 2.5 mg at 4 pm). She lost 9 kg during this period, maintained normal blood pressure, fasting glucose levels < 80 mg/dL, and remission of CS. A timeline for this case report is provided in Fig. 3.

## Discussion

CS presentation can be quite variable among adults and children, depending on the duration and the severity of hypercortisolemia. Nonetheless, typical signs and symptoms of CS are generally well recognized [13]. However, rarely patients presenting with unusual features can go unnoticed, leading to a delay in diagnosis and treatment of this condition, which can be deleterious. Avascular necrosis of the hip associated with CS has been described in a few patients [2–12], and previous reported cases are summarized in Table 2. A total of 14 cases were reported, 79% female (*n*=11), with a mean age of 38 ± 13.5 years, and 57% being of pituitary origin. This distribution is equal to the general CS characteristics.

**Table 2:** Characteristics of clinical cases of Cushing syndrome with avascular necrosis

Author	No. of cases	Sex	Age	Etiology	Treatment
Phillips *et al*. [2]	4	Woman	24 25 43 61	Cushing’s disease	Transsphenoidal hypophysectomy
Belmahi *et al*. [3]	1	Woman	28	Cushing’s disease	Transsphenoidal hypophysectomy
Koch *et al*. [4]	1	Woman	30	Cushing’s disease	Transsphenoidal hypophysectomy
Premkumar *et al*. [5]	1	Woman	26	Cushing’s disease	Transsphenoidal hypophysectomy
Wicks *et al*. [6]	1	Man	39	Cushing’s disease	Craniotomy with hypophysectomy
Saeed *et al*. [7]	1	Woman	20	Adrenal nodular cortical hyperplasia	Right adrenalectomy
Takada *et al*. [8]	1	Woman	55	Adrenal adenoma	Adrenalectomy
Ha *et al*. [9]	1	Woman	36	Adrenal cortical adenoma	Left partial adrenalectomy
Pazderska *et al*. [10]	1	Woman	36	Primary pigmented micronodular adrenal disease	Left plus right adrenalectomy
Cerletty *et al*. [11]	1	Man	54	Bilateral adrenal cortical hyperplasia	Bilateral adrenalectomy
Camporro *et al*. [12]	1	Man	55	Ectopic Cushing’s syndrome	Pulmonary segmentectomy

Many factors, both traumatic and nontraumatic, contribute to the occurrence of avascular bone necrosis, but the use of exogenous glucocorticoids and alcoholism are among the most common nontraumatic causes [1, 14]. The pathogenic mechanisms of avascular necrosis of the femoral head caused by endogenous hypercortisolism or exogenous glucocorticoid excess are not fully understood, but it has been proposed that fat cell hypertrophy, fat embolization, and apoptosis of osteocytes cause compromise of blood perfusion in the femur, leading to ischemic necrosis of the tissues [14]. Another suggested hypothesis is that hypertension and atherosclerosis secondary to excess glucocorticoids may induce insulin resistance and lead to avascular necrosis [15]. Factors related to pregnancy might predispose a susceptible patient to adverse bone events [16]. Excessive weight gain, especially in the third trimester, and mechanical stress or micro-trauma during delivery may have contributed to trigger femoral avascular necrosis in our patient. The high prevalence of female cases in the literature (79%) seems to support this conception, and it would be of interest to better explore the role of pregnancy/delivery in the occurrence of bone avascular necrosis.

Early diagnosis of this condition is crucial for optimal treatment, directed toward the etiology of the disease, because the process is often progressive, resulting in joint destruction within 3 to 5 years if left untreated, with the need for hip replacement in up to 70% of patients [14]. The majority of patients described with avascular bone necrosis due to endogenous hypercortisolism were treated surgically with core decompression, osteotomy, or total hip replacement, as in our patient. However, spontaneous resolution of avascular necrosis of femoral heads after bilateral adrenalectomy for CS caused by primary pigmented nodular adrenocortical disease has been described [10], and conservative treatment for stages 1–4 has been supported in the recent literature, although stage 3 and 4 patients might need hip replacement surgery [17, 18].

Our case demonstrates that avascular necrosis of the hip may be an alarming presenting feature of CS. Rare causes for osteonecrosis, such as endogenous hypercortisolism, should be suspected in every patient who presents with avascular necrosis of the hip with unknown etiology (no risk factors for bone ischemia or history of trauma). Our patient was a young woman with early onset of HBP and a 3-year history of leg pain and decreased mobility, with a late diagnosis of avascular bone necrosis. Also, it was only 2 years after hip arthroplasty, with the emergence of secondary amenorrhea, that she was properly referred for evaluation by an endocrinologist, showing marked cushingoid stigmata. Earlier identification of osteonecrosis and of CS might have prevented bone collapse and the need for an arthroplasty at a young age—with risk for revision in the future—and might have prevented the occurrence of DVT, probably secondary to the hypercoagulability state associated with hypercortisolism.

Further research on the mechanisms of bone necrosis might be helpful in understanding how and why avascular necrosis develops in some patients with endogenous hypercortisolism.

## Conclusions

Avascular bone necrosis associated with CS is a rare occurrence. It happens mostly in women, and has a pituitary origin in more than half of patients. Many other factors might concomitantly contribute to its occurrence.

As this presentation is rare, our case reinforces the importance of a high index of suspicion and early recognition of CS atypical features, in order to avoid complications and delay in treatment of CS.

## Data Availability

All data generated or analysed during this study are included in this published article.

## Abbreviations

ACTH: Adrenocorticotropic hormone
BMI: Body mass index
CS: Cushing syndrome
CT: Computed tomography
DHEA-S: Dehydroepiandrosterone sulfate
DST: Dexamethasone suppression test
DVT: Deep venous thrombosis
FSH: Follicle-stimulating hormone
FT4: Free thyroxine
FT3: Free triiodothyronine
HbA1c: Glycated hemoglobin
HBP: High blood pressure
IGF-1: Insulin-like growth factor 1
LH: Luteinizing hormone
MRI: Magnetic resonance imaging
PRL: Prolactin
SHBG: Sex hormone-binding globulin
TSH: Thyroid-stimulating hormone
UFC: Urinary free cortisol
