# Correction to: Avascular femoral necrosis as part of Cushing syndrome presentation: a case report

**DOI:** 10.1186/s13256-021-03105-9

**Published:** 2021-09-23

**Authors:** Daniela Salazar, César Esteves, Maria João Ferreira, Jorge Pedro, Tiago Pimenta, Raquel Portugal, Davide Carvalho

**Affiliations:** 1 Department of Endocrinology, Diabetes and Metabolism, Centro Hospitalar Universitário de São João, 4200-319 Porto, Portugal; 2 Faculty of Medicine of University of Porto, Porto, Portugal; 3 Institute for Research and Innovation in Health, University of Porto, Porto, Portugal; 4 Department of Surgery of Centro Hospitalar, Universitário de São João, Porto, Portugal; 5 Department of Pathology of Centro Hospitalar, Universitário de São João, Porto, Portugal

## Correction to: J Med Case Reports (2021) 15:287 https://doi.org/10.1186/s13256-021-02882-7

Following publication of the original article [1], the authors identified an error in the author name of Davide Carvalho.

The incorrect author name is: David Carvalho.

The correct author name is: Davide Carvalho.

The author group has been updated above and the original article [1] has been corrected.
